# Correction: A circuit model of auditory cortex

**DOI:** 10.1371/journal.pcbi.1010700

**Published:** 2022-11-15

**Authors:** Youngmin Park, Maria N. Geffen

In the sub-section ‘Three-unit Rate model’ of the section ‘Materials and methods’, there is a typographical error in equation (6). Please view the complete, correct equation here:

$$J_{1,i}(t)=\left\{ \begin{array}{c} -F_{i}(t)s_{2}(t)+qI_{i}(t)+w_{ee}^{*}u_{2}(t)/1.5\\ -F_{2}(t)(s_{1}(t)+s_{3}(t))+{qI}_{2}(t)+\frac{w_{ee}^{*}(u_{1}(t)+u_{3}(t))}{2} \end{array}\right.$$

In the sub-section ‘Differential effects of inhibitory neuron manipulation on cortical forward suppression’ of the section ‘Results’, there are some typographical errors in the sixth sentence of the second paragraph. The correct sentence is:

In the rate model, the inhibition was dimensionless and free parameters chosen to be *I*_Opt,PV_(*t*) = 0.1 during PV inhibition, *I*_Opt,PV_(*t*) = 0.025 during PV activation, *I*_Opt,SST_(*t*) = −0.5 during SST inhibition, and *I*_Opt,SST_(*t*) = 0.1 during SST activation.

There is a typographical error in Table 1. The value for *I*_Opt,PV_(*t*) in the column ‘FWS’ should be -0.1 (0.025). Please view the corrected table here.

**Table 1:** Parameter values of rate models across paradigms.

Parameter	SSA (Simple)	SSA	FWS	TCA	PV Act.	Notes
q	5	5	1.3	5	5	Fitted, dimensionless, rate
*I*_Opt,PV_(*t*)	-2	−4 (0.5)	-0.1 (0.025)	-0.5 (1.2)	2 (2)	Fitted, dimensionless, rate
*I*_Opt,SST_(*t*)	-1	-2 (1.2)	-0.5 (0.1)	-1 (0.1)	-	Fitted, dimensionless, rate
q	5	5	1.3	5	5	Fitted, dimensionless, spiking
*I*_Opt,PV_(*t*)	-	-0.2nA	0.1nA	1nA	0.5nA	Fitted, dimensionless, spiking
*I*_Opt,SST_(*t*)	-	-1nA	2nA	1nA	-	Fitted, dimensionless, spiking

In the optogenetic parameters, numbers without parentheses show optogenetic strengths for use in model reproductions and numbers in parentheses show optogenetic strengths for use in model predictions. Positive optogenetic numbers correspond to activation and negative numbers correspond to inactivation.
